# Association of serum retinoic acid with depression in patients with acute ischemic stroke

**DOI:** 10.18632/aging.102767

**Published:** 2020-02-10

**Authors:** Cai-Di Yang, Ming-Li Cheng, Wen Liu, Ding-Hua Zeng

**Affiliations:** 1 Department of Neurology, Eastern Hospital, Sichuan Academy of Medical Sciences and Sichuan Provincial People's Hospital, Chengdu 610101, China; 2 Department of Neurology, People's Hospital of Jianyang, Jianyang 641400, China; 3 The Clinical Laboratory Department, Affiliated Hospital of North Sichuan Medical College, Nanchong 637000, China

**Keywords:** retinoic acid, stroke, ischemic stroke, depression

## Abstract

Retinoic acid (RA), produced by the metabolism of vitamin A, makes effects on depression and stroke. This study was aimed to evaluate the relationship between RA levels in serum and post-stroke depression (PSD). A single-center (Chengdu, China) prospective cohort study was conducted on patients with acute ischemic stroke. The RA serum level was measured at admission. The PSD was assessed in the 3-month follow-up. The RA-PSD relationship was evaluated with conditional logistic regression. In total, 239 ischemic stroke cases and 100 healthy controls were included. The median RA serum level in patients with ischemic stroke was 2.45 ng/ml (interquartile range [IQR], 0.72-4.33), lower(P<0.001) than 3.89 ng/ml of those in control cases ([IQR]: 2.62-5.39). The crude and adjusted odds ratios [OR] (and 95% confidence intervals [CI]) of PSD associated with an IQR increase for RA were 0.54 (0.44, 0.67) and 0.66 (0.52, 0.79), respectively. Higher ORs of PSD associated with reduced RA levels (<cut-off=2.8ng/ml) were observed (OR=3.01 [95% CI, 2.34-4.98]; P<0.001). This study revealed that, in patients with ischemic stroke, reduced RA serum level was related to higher risk of PSD at 3 months, which may be applied as a predictive indicator.

## INTRODUCTION

Stroke has been a leading cause of disability and death worldwide [1]. In China, stroke also is associated with the highest disability-adjusted life-years loss of any disease, and with over 2 million new cases annually [2]. Since China contains a fast ageing population, it has been a challenge to prevent and control stroke as well as its complications [3].

Post-stroke depression (PSD), which is an important psychosocial consequence after stroke associated with higher stroke morbidity, mortality, and recurrence, affects one in third of patients [4]. A meta-analysis showed that PSD significantly increased risk of death in stroke patients [5]. Psychopathological mechanism of PSD has been complex and unclear, which may be associated with the neurobiological dysfunctions induced by ischemic stroke, such as the neuroinflammatory caused by ischemia, stress activation of the hypothalamic-pituitary-adrenal (HPA) axis, as well as the dysfunction of adaptive response [6].

Retinoic acid (RA), produced by the metabolism of vitamin A, makes effects on cell growth, differentiation, organogenesis, and mucosal immune responses [7, 8]. Some studies had reported that RA made regulatory effects on cardiac development and protective effects on vascular systems [9, 10]. Liu et al. [11] revealed that serum RA level was related to the reduced risk of death in patients with coronary artery disease. Furthermore, low circulating levels of RA were reported to link with uplifted risk of mortality in ischemic stroke [12].

Nucleus accumbens shell is crucial for depression and one study reported that RA signaling pathway was enhanced in this region [13], which influenced on the emotional behavior, such as depression and anxiety [14]. Accumulating studies have showed an association between RA and depression development [15–17]. However, the relationship between RA and PSD has not elucidated. We conducted a prospective single-center cohort study on patients with acute ischemic stroke (AIS) for evaluating the relationship between RA serum levels and PSD, and the clinical significance of RA was also compared with other known PSD indicators.

## RESULTS

### Basic information

**Figure 1:**
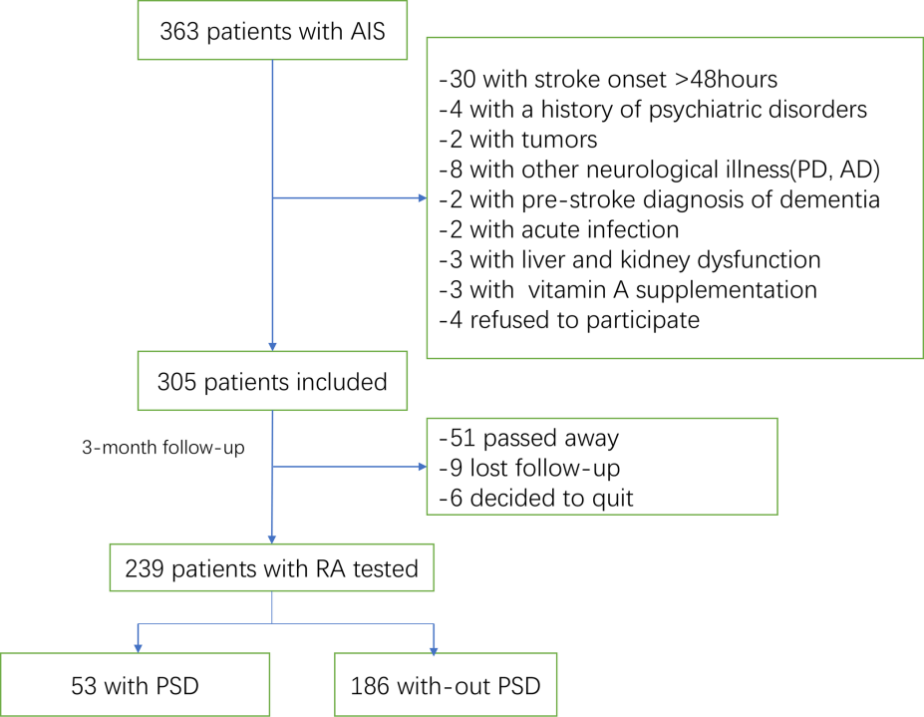
**Study flow diagram.**

363 patients with AIS were recruited. As shown in the Figure 1, 305 patients were included. In the follow-up, 51 patients had passed away and 15 patents had lost follow-up or decided to quit, leaving 239 individuals (Figure 1). For all the included patients, 138(57.7%) were male, with median age of 65(IQR, 56-77). At admission, the National Institutes of Health and Stroke Scale (NIHSS) score (median [IQR]) was 7 [4–11], and 31(13.0%) patients received thrombolytic therapy. The median RA serum level in patients with ischemic stroke was 2.45 ng/ml (interquartile range [IQR], 0.72-4.33), lower (P<0.001) than 3.89 ng/ml of those in control cases ([IQR]: 2.62-5.39).

### Main results

For the 363 patients with AIS, 53 patients were diagnosed as depression at 3-month follow-up, showing the incidence rate was 22.2% (95%CI: 16.9%-27.4%). The characteristics of the patients with or without depression were presented (Table 1). Compared with those without PSD, PSD patients showed higher age, ratio of female and widowhood, stroke severity and 3-month functional disability, as well as higher serum levels of Hs-CRP and HCY.

**Table 1:** Clinical characteristics in patients with and without PSD^†^.

	**ALL**	**PSD**	**No-PSD**	**P**
No	239	53	186	-
Age	65(56-77)	69(60-81)	63(53-73)	0.009
Sex-male	138(57.7)	24(45.3)	114(61.3)	0.037
BMI	24.8(22.9-26.5)	25.0(23.1-26.4)	24.7(22.8-26.5)	0.12
Education	12(9-15)	12(9-15)	12(9-15)	0.85
Hypertension	168(70.3)	36(67.9)	132(71.0)	0.67
Diabetes Mellitus	63(26.4)	15(28.3)	48(25.8)	0.72
Coronary heart disease	52(21.8)	12(22.6)	40(21.5)	0.86
Family history of stroke	25(10.5)	7(13.2)	18(9.7)	0.46
Family history of psychiatric disorders	16(6.7)	7(13.2)	9(4.8)	0.032
Widowhood or divorced	19(7.9)	9(17.0)	10(5.4)	0.006
Time from stroke onset to blood collected	12.0(7.0-25.0)	13.5(8.0-28.0)	11.5(7.0-24.0)	0.029
Stroke etiology				0.59
Large-artery atherosclerosis	80(33.5)	21(39.6)	59(31.7)	
Cardio-embolism	55(23.0)	12(22.6)	43(23.1)	
Small vessel disease,	47(19.7)	9(17.6)	38(20.4)	
Other	24(10.0)	5(9.4)	19(10.2)	
Unknown	33(13.8)	6(11.3)	27(14.5)	
Stroke location				0.37
lobe	24(10.0)	6(11.3)	18(9.7)	
Thalamus	21(8.8)	7(13.2)	14(7.5)	
Brainstem	46(19.2)	10(18.9)	36(19.4)	
Basal ganglia or lateral ventricles	116(48.5)	23(43.4)	93(50.0)	
Cerebellum	6(2.5)	2(3.8)	4(2.2)	
Multiple locations	26(10.9)	5(9.4)	21(11.3)	
NIHSS at admission	7(4-11)	9(6-12)	6(3-10)	0.002
mRS at discharge	1(1-2)	2(1-3)	1(1-2)	0.015
HAMD at 3-month	4(2-7)	3(1-4)	10(7-13)	<0.001
Laboratory testing ^††^				
Fasting serum glucose, mmol/l	5.75(5.11-6.25)	5.89(5.21-6.43)	5.59(4.90-6.04)	0.013
Hs-CRP, mg/dl	0.61(0.24-1.25)	0.68(0.28-1.48)	0.50(0.22-1.14)	<0.001
HCY, umol/l	15.5(11.9-19.7)	16.8(13.5-21.4)	14.5(10.4-18.6)	0.008
RA, ng/ml	2.45(0.72-4.33)	1.27(0.46-2.75)	2.94(0.99-4.64)	<0.001

^**†**^Results were expressed as numbers(percentages) for categorical variables or as medians (IQR) for the continuous variables.

^††^ Serum levels of fasting serum glucose, Hs-CRP, HCY and RA in matched controls (N=100) were 5.15(4.85-5.68) mmol/l, 0.22(0.13-0.35) mg/dl, 12.5(10.8-16.7) umol/l and 3.89(2.62-5.39) ng/ml, respectively.

PSD, Post-stroke depression; NIHSS, National Institutes of Health Stroke Scale; mRS, Modified Rankin Scale; IQR, interquartile range; CRP, C-reactive protein; HCY, homocysteine; RA, Retinoic acid; BMI, body mass index; HAMD, Hamilton Rating Scale for Depression.

**Figure 2:**
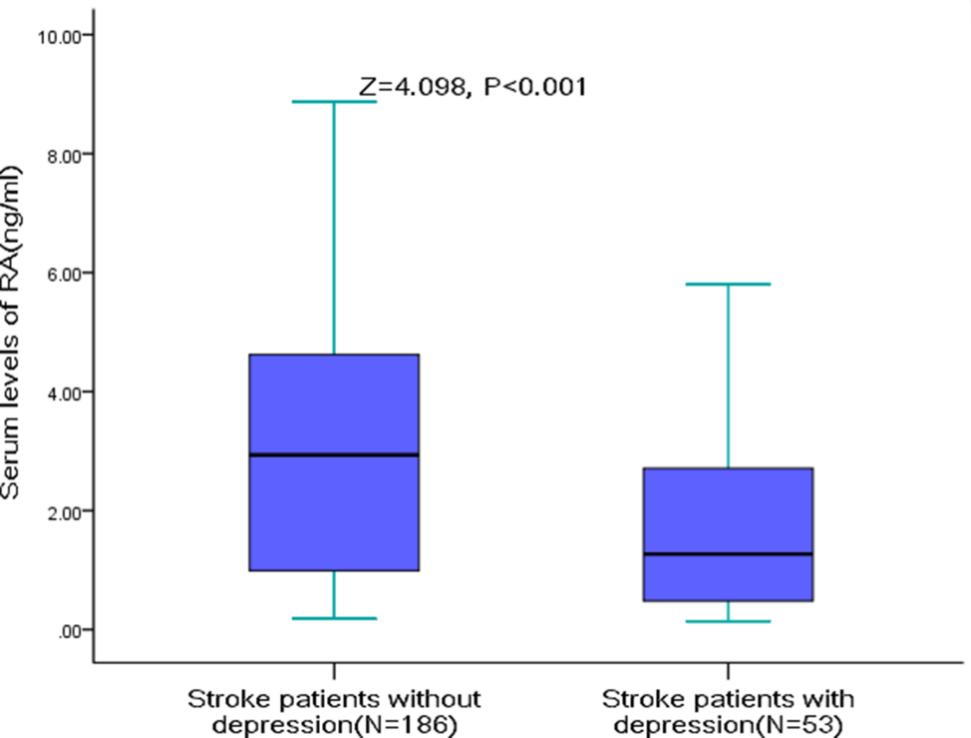
**RA Serum levels in stroke patients with PSD and without PSD.** All data are medians and inter-quartile ranges (IQR); P values refer to Mann-Whitney U tests for differences between groups. PSD=Post-stroke depression; RA=Retinoic acid.

The serum RA levels were significantly lower in AIS patients with PSD (median [IQR]: 1.27[0.46-2.75] vs. 2.94[0.99-4.64]; Z=4.098, P<0.001, Figure 2). The serum RA level was negatively correlated with the severity of depression (r[spearman]=-0.249, P<0.001), which was defined with HAM-D score. Furthermore, negative correlations were observed between RA serum level and the NIHSS score (r=-0.175, P=0.007), Hs-CRP serum levels (r =-0.209, P=0.001) HCY serum levels (r=-0.132, P=0.041) and age (r =-0.184, P=0.005). The RA serum level was not fluctuated with the blood collection time from stroke onset (P=0.17).

The ORs and 95% CIs of PSD associated with an IQR increase in RA were compared with other risk indicators including the NIHSS score (Table 2). Higher serum levels of RA significantly decreased the risk of PSD. The crude OR (and 95% CI) was 0.54(0.44, 0.67). The OR was adjusted for potential confounders and the adjusted OR (95% CI) was 0.66(0.52, 0.79). In addition, age, sex, widowhood, higher initial stroke severity, 3-month functional disability and serum levels of Hs-CRP and HCY were significant indicators for depression, different from others factors (Table 2). Furthermore, multivariate analysis was performed to obtain adjusted OR (95% CI) of PSD associated with RA quartiles (with Q1 as reference) are shown in Table 3. PSD distribution for the RA quartiles ranged from 36.7% (Q1) to 8.5% (Q4) (P <0.001). RA serum levels in Q3 and Q4 were related to less risk of PSD, which was decreased by 48% (OR=0.52; 95%CI: 0.36-0.92; P=0.039) and 67% (OR=0.36; 95%CI: 0.18-0.63; P<0.001).

**Table 2:** Conditional logistic regression models were used to estimate the associations between RA, NIHSS score, other risk factors and PSD.

**Parameter**	**Univariate Analysis**		**Multivariate Analysis^‡^**	
	**Crude ORs (95% CIs)**	**P**	**Adjusted ORs (95% CIs)**	**P**
Age (per unit increase)	1.07(1.04-1.09)	0.009	1.05(1.01-1.10)	0.012
Sex (male vs. female)	0.53(0.28-0.97)	0.04	0.64(0.35-1.00)	0.05
BMI (per unit increase)	0.90(0.80-1.03)	0.12		
Education (per unit increase)	1.03(0.90-1.15)	0.85		
Hypertension (Yes vs. no)	0.87(0.45-1.67)	0.67		
Diabetes Mellitus (Yes vs. no)	1.14(0.57-2.25)	0.72		
Coronary heart disease (Yes vs. no)	1.07(0.52-2.22)	0.86		
Family history of stroke (Yes vs. no)	1.42(0.56-3.61)	0.46		
Family history of psychiatric disorders (Yes vs. no)	2.99(1.06-8.46)	0.03	2.16(1.01-6.15)	0.04
Widowhood or divorced (Yes vs. no)	3.60(1.38-9.60)	0.006	3.04(1.25-9.03)	0.02
Time from stroke onset to blood collected	1.17(1.05-1.31)	0.029	1.08(0.97-1.28)	0.08
Stroke etiology				
Large-artery atherosclerosis	0.97(0.47-2.02)	0.94		
Cardio-embolism	1.41(0.75-2.66)	0.42		
Small vessel disease,	0.80(0.46-1.77)	0.58		
Other	0.92(0.33-2.58)	0.87		
Unknown	0.75(0.29-1.93)	0.55		
Stroke location				
lobe	1.19(0.45-3.17)	0.73		
Thalamus	1.87(0.72-4.92)	0.20		
Brainstem	0.97(0.45-2.11)	0.94		
Basal ganglia or lateral ventricles	0.77(0.42-1.42)	0.77		
Cerebellum	1.78(0.32-10.02)	0.51		
Multiple locations	0.82(0.29-2.29)	0.70		
NIHSS at admission (per unit increase)	1.07(1.04-1.10)	0.002	1.04(1.01-1.09)	0.009
mRS at discharge (per unit increase)	1.24(1.13-1.32)	0.015	1.15(1.07-1.29)	0.04
Laboratory testing				
Fasting serum glucose (per unit increase)	1.15(1.03-1.27)	0.013	1.08(1.01-1.23)	0.04
Hs-CRP (per unit increase)	1.38(1.16-1.63)	<0.001	1.25(1.09-1.51)	0.002
HCY (per unit increase)	1.04(1.01-1.07)	0.012	1.02(1.00-1.06)	0.04
RA (per IQR increase)	0.54(0.44-0.67)	<0.001	0.66(0.52-0.79)	<0.001

^‡^Factors adjusted in multivariate analysis were defined in the univariate analysis, including age, sex, family history of psychiatric disorders (yes vs. no), widowhood or divorced (yes vs. no), time from stroke onset to blood collected, NIHSS at admission, mRS at discharge, serum levels of glucose, Hs-CRP, HCY and RA.

PSD, Post-stroke depression; NIHSS, National Institutes of Health Stroke Scale; mRS, Modified Rankin Scale; IQR, interquartile range; CRP, C-reactive protein; HCY, homocysteine; RA, Retinoic acid; BMI, body mass index; OR, odd ratio; CI, Confidence Interval.

**Table 3:** Multivariate analysis to estimate adjusted OR (95% CI) of PSD associated with RA quartiles (with Q1 as reference).

**RA quartiles (No.)†**	**No. of PSD (%)**	**Univariate Analysis**		**Multivariate Analysis‡**	
		**Crude ORs (95% CIs)**	**P**	**Adjusted ORs (95% CIs)**	**P**
Q1(60)	22(36.7)	Reference	-	Reference	-
Q2(60)	16(26.7)	0.63(0.29-1.37)	0.24	-	-
Q3(60)	10(16.7)	0.35(0.15-0.82)	0.013	0.52(0.36-0.92)	0.039
Q4(59)	5(8.5)	0.16(0.06-0.48)	<0.001	0.36(0.18-0.63)	<0.001

†RA quartiles were defined as Q1: <0.72ng/ml; Q2: (0.72-2.45) ng/ml; Q3: (2.46-4.33) ng/ml; Q4: >4.33 ng/ml.

‡Factors adjusted in multivariate analysis were defined in the univariate analysis, including age, sex, family history of psychiatric disorders (yes vs. no), widowhood or divorced (yes vs. no), time from stroke onset to blood collected, NIHSS at admission, mRS at discharge, serum levels of glucose, Hs-CRP, HCY and RA.

PSD, Post-stroke depression; NIHSS, National Institutes of Health Stroke Scale; mRS, Modified Rankin Scale; IQR, interquartile range; CRP, C-reactive protein; HCY, homocysteine; RA, Retinoic acid; OR, odd ratio; CI, Confidence Interval.

**Figure 3:**
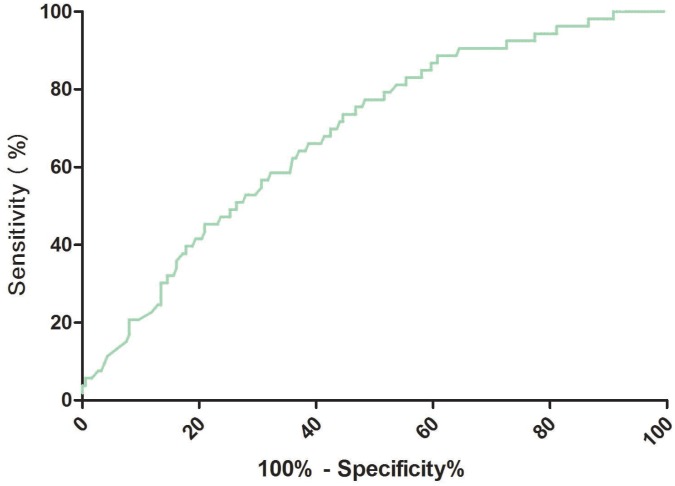
**Receiver operator characteristic curve demonstrating sensitivity as a function of 1-specificity for predicting the PSD based on the RA serum levels.** PSD= Post-stroke depression; RA=Retinoic acid.

The cut-off value of RA for predicting PSD was calculated from ROC curves. When the RA serum level was 2.8ng/ml, the AUC was 0.69 (95% CI, 0.61-0.76), providing a best sensitivity of 77.4% and specificity of 51.6% (Figure 3). RA presented a better discriminatory ability than that of age (AUC, 0.58; 95%CI, 0.50-0.64; P=0.001), sex (AUC, 0.55; 95%CI, 0.47-0.61; P<0.001) and was within the range of the NIHSS score (AUC, 0.71; 95%CI, 0.63-0.78; P=0.42). In addition, RA was superior to other serum indicators, including Hs-CRP (AUC, 0.62; 95% CI, 0.56–0.69; P=0.006), HCY (AUC, 0.63; 95% CI, 0.58–0.70; P=0.012), and glucose (AUC, 0.52; 95% CI, 0.42–0.58; P<0.001). A multivariate model was constructed with reduced levels of RA (<cut-off=2.8ng/ml) combining with the other factors and the results showed predictive significance (PSD: OR=3.01 [95% CI, 2.34-4.98]; P<0.001]).

## DISCUSSION

Previous studies have confirmed that PSD was caused by multiple factors, conformed to the biopsychosocial model of mental illness [18]. RA functioned in the development of the central nervous system [19]. In our prospective cohort study, the RA was proved as a new and independent predictor for indicating 3-month PSD in ischemic stroke patients. It was compatible with the existing indicator of clinical NIHSS score, while superior to that of other indicators.

Consistent with this study, Duan et al. [20] reported that decreased serum levels of RA at baseline were related to the risk of 3-month depression in AIS patients and Hou et al. [21] showed that reduced RA serum level was associated with 3-month poststroke cognitive impairment (PSCI) in AIS patients. PSD might occur through a pathophysiological pathway other than classical depression [22]. PSD may greatly influence the prognosis of stroke [23], both depression symptoms and cognitive impairment were independent predictors of impaired higher-level functioning and community reintegration 2 to 3 years after stroke [24]. A study showed that PSD made negative effects on functional outcome, despite of pharmacological therapy [25].

Various parameter would be tested on admission, some of which were probably associated with PSD, for. instance, vitamin D [26], vitamin B [27], insulin-like growth factor 1 [28], interleukin-6 [29]; hs-CRP [30], neopterin [31], tumor Necrosis Factor–α [32], and irisin [33]. Timely and accurate risk assessment was useful for depression prevention and care of stroke patients [34]. Our study illustrated that serum RA level was additionally helpful for predicting the 3-month PSD in AIS patients.

Significant association of RA level and age in PSD patients had been found. This is not surprising that the absorption of nutrients such as vitamin A decrease among older patients. Thus, the age would contribute to the association between RA and PSD. However, this relationship persists after adjusting for age in multivariate analysis. Furthermore, patients with depression was accompanied with higher infarct volumes and severer functional disability. RA levels were negatively correlated with the functional disability degree. The association between RA and PSD might be resulted from the stroke severity and disability. Again, correlation still persists after adjusting for these factors in multivariate analysis. Thus, the role of RA in pathophysiology of PSD might be functioned through the following pathways. First, RA played role in both the innate and adaptive immune systems [8], thus regulating the brain function at the cellular and systemic levels, finally functioned in pathology of depression [35]. Second, RA promoted the induction of proinflammatory cytokines and the maintenance of tissue homeostasis after the brain ischemia, finally contributing to the pathogenesis of mood disorders [36, 37]. In mice with chronic inflammation in the ileum, oral supplementation with RA could alleviate inflammation by regulating the balance between the Th17 and Treg populations. In addition, the number of CD103+ DCs and RALDH2 expression would be enhanced via a positive feedback mechanism [38, 39]. Third, neurovascular unit dysfunction with blood–brain barrier (BBB) hyperpermeability contributed to depressive disorder [40]. In an in vitro oxygen–glucose deprivation (OGD) treatment, RA made protective effects on BBB, depending on RA receptor α [41]. Fourth, increased oxidative stress in cerebral tissues during ischemia had been proposed to implicate in the pathogenesis of depressive-like symptoms following stroke [42]. One study showed that RA reduced staurosporine-induced oxidative stress and apoptosis and supported the antioxidant defense system [43]. One study reported that all-trans-RA (at-RA) increased nitric oxide (NO) synthesis in endothelial cells [44]. NO mechanism in the protective effect of naringin against PSD in mice had been recommended [45]. Fifth, bone morphogenetic protein (BMP) signaling in the hippocampus regulated depression, suppressed BMP signaling involved in the effects of some antidepressants [46]. 9c-RA had protective effects against ischemic brain injury, and these effects involve BMP [47]. Lastly, RA and RA receptor (RAR) and their complex play roles in innate and adaptive immunity [48]. In addition, RA showed neuroprotective activity, which could increase barrier tightness in human-induced pluripotent stem cell-derived brain endothelial cells by activating RARα, RARγ, and RXRα [49]. RAR plays both sides of homeostatic plasticity [50], and Chiang et al., [51] showed that RAR contributed to long-term depression, revealing a novel and unexpected role for vitamin A in higher cognitive functions. RARα was implicated in the activation of HPA axis thus participating in the etiology of depression [52].

Observative research design could not clarify the relationship between the RA, depression and outcomes in stroke patients. Machado-Pereira et al. [53] suggested that systemic administration of RA-loaded nanoparticles protected neurovascular integrity and improved the inflammatory environment from ischemia in the immature brain, demonstrating the effects of RA in the regulation of neuroinflammation. Plane et al. [54] showed that the RA could improve the survival of adult-generated subventricular zone-derived neurons and stimulate post-stroke function recovery. 9-cis-RA had neuroprotective effects against ischemia-related brain injury [55]. At-RA showed potent therapeutic efficacy in ischemic stroke by attenuating neural inflammation through STAT1 signaling [56]. In cerebral ischemia rats, the infarct volume could be reduced with RA, and the neural functional recovery would be enhanced [19]. The role of serum RA during stroke, and whether supplemented RA could prevent depression to improve stroke prognosis need further experimental validation.

### Study strengths and limitations

This study included first-ever AIS patients admitted 48 h within the onset of symptoms. These samples were more representative of the acute state. Furthermore, a range of confounding risk factors had been collected and adjusted. Lastly, we used the method of interquartile range in statistical analysis.

There were still some notable limitations. First, this is a preliminary study with a small sample size (N=239) from one single center. The conclusion could not be over-interpreted. Second, patients with a lifespan of less than three months and aphasia were excluded, and these patients might be suffered from depressive symptoms. This might cause a selection bias, resulting in a lower rate of PSD than actual data. Third, the PSD patients' quality of life was not assessed in this study. Fourth, serum level of RA was tested once at admission, thus the changes in the disease course could not be monitored. RA levels were assessed without previous vitamin A and/or base treatment, which may affect RA determination. Thus, the results should be verified in the future. Furthermore, we tested serum rather than cerebral spinal fluid RA levels, thus RA levels in the central nervous system could not been determined. Lastly, this was an observational cohort study which could not draw any causal relationship. Whether maintaining normal RA serum level favored for PSD prevention should be further investigated.

In conclusion, reduced RA serum level was correlated to higher risk of 3-month depression in AIS patients, suggesting that RA may be a promising predictive biomarker for PSD. This may be used for improving prediction of depression and assignment of effective treatment for stroke in the future.

## MATERIALS AND METHODS

### Ethical statement

This study was reviewed and approved by the Patient Research Ethics Committee of the Sichuan Provincial People's Hospital (Permission Numbers: 2016-03-18A) and conducted strictly adhered to the principles of the Declaration of Helsinki. Written informed consents were obtained from all participants or from their relatives (e.g., paralysis or blindness).

### Included patients

This single-center prospective cohort study was conducted in Chengdu, China. From September 1, 2017 to October 30, 2018, all patients were first-ever AIS who were admitted to the Department of Emergency of our hospital. AIS was confirmed according to the criteria of WHO Multinational Monitoring of Trends and Determinants in Cardiovascular Disease (WHO-MONICA) [57]. The inclusion criteria included: (1) stroke was diagnosed based on magnetic resonance imaging (MRI) and/or computerized tomography (CT). (2) symptom onset time were within 48hours. Furthermore, the exclusion criteria was as follows: (1) severe aphasia and/or cognitive impairment, decreased level of consciousness and dementia; (2) severe neurological illness (e.g., Parkinson’s disease, Alzheimer Disease); (3) other medical illness (e.g., cancer, acute infection and liver and/or renal insufficiency); (4) a history of depression or other psychiatric disorders; (5) survival period < 3 months; (6) current use of vitamin A supplementation.

At the same time, 100 healthy volunteers with matched age and sex were recruited in control group from hospital medical examination center. Exclusion criteria for patients, was also applied to the control group. In addition, the psychiatric or cerebrovascular diseases were excluded by a neurological doctor (not the author). The Hamilton Scale in those subjects should less than 7.

### Clinical variables at admission

At admission, the basic information (age, sex, education and BMI), vascular risk factors (hypertension, diabetes mellitus, coronary heart disease and family history of stroke), family history of psychosis and marital status were recorded. Stroke severity was analyzed by skilled neurologists according to the NIHSS score [ranged from 0 to 42]; higher score indicated severer disease) [58]. MRI was used to evaluate the site and cause of stroke within 24hours after admission. Stroke etiology was evaluated with the TOAST (Trial of Org 10172 in Acute Stroke Treatment) criteria [59]. In addition, functional outcome was achieved at discharge based on the modified Rankin Scale (mRS) blinded to RA serum levels [60].

### Assessment of depression

The depression was the primary end point of AIS patients in the 3-month follow-up. The depression was defined by the Diagnostic and Statistical Manual of Mental Disorders IV (DSM-IV) criteria. Patients with a diagnosis of PSD must show depressed mood or loss of interest or pleasure, accompanied with 2~5 symptoms of major depression lasted for at least 2 weeks [61]. In addition, the validated Chinese translation Hamilton Depression Rating Scale 17 items (HDRS, also abbreviated HAMD) was also used to quantify the severity of depressive symptoms [62]. Cut-off threshold for depression were chosen as >7 points for each of the HDRS [63]. Good internal consistency of HDRS was observed in the patients included in our study (Cronbach's α for HDRS-was 0.82). During the follow-up, antidepressant medication will be given immediately if the patients were diagnosed as PSD. The all-cause mortality within the 3-month follow-up was the secondary end point. Outcome assessment was performed by two skilled physician who were blind to the patients’ information. A structured telephone interview was performed on the patient or their relatives.

### Blood sampling and biomarkers testing

Fasting venous blood of patients was collected on admission, within 72 h after the onset of symptom (within 0–3[n=25], 3–12 [n=94], 12–24 [n=52], and 24–72[n=68]). The serum was obtained by centrifuge at 3000 g and then frozen at −70 °C until testing. The RA serum level was quantified with ELISA kit (catalog no. MBS705877; MyBio-Source). Previous studies had suggested the high sensitivity and specificity of this method in the determination of human RA [12, 64]. This kit showed slight cross-reactivity with vitamin B-12(within 10%), vitamin D (within 8%) and vitamin A (within 10%). The coefficient of variance for the inter-assay and intra-assay repeatability was less than 10% and 8%, respectively. In addition, the serum levels of other parameters were also tested with standard methods, including homocysteine (HCY), hypersensitive C-reactive protein (Hs-CRP) and fasting blood glucose (FBG).

### Statistical analysis

The normal distribution of data was evaluated with Kolmogorov-Smirnov test. The categorical variables and continuous variables were expressed as numbers(percentages) or edians (interquartile range, IQR), respectively. The comparison among groups was performed with Student's t-test or Mann-Whitney's U test. Correlation was assessed with Spearman's correlation coefficient.

The correlation between PSD and RA serum level was evaluated with conditional logistic regression models. A range of risk factors of PSD and potential confounders were confirmed according to univariate analysis. We adjusted the confounders in the model, including age, sex, family history of psychosis (yes vs. no), widowhood or divorced (yes vs. no), NIHSS at admission, time from stroke onset to blood collection, mRS at discharge, FBS, Hs-CRP, HCY and RA. The association between RA and PSD were presented as odds ratio (OR) and 95% confidence intervals (95% CI) associated with an interquartile range (IQR) increase. Furthermore, multivariate analysis was also used to obtain adjusted OR (95% CI) of PSD associated with RA quartiles (with Q1 as reference).

Accuracy of RA serum level and other factors to predict PSD was evaluated with ROC. Optimal RA serum level in the ROC was confirmed with the level with the highest Yuden index, for predicting depression symptoms after stroke. The effects of reduced levels of RA (<cut-off value, defined by ROC analysis) on PSD was also assessed. Statistics was performed with SPSS 23.0 (SPSS Inc., Chicago, IL, USA) and GraphPad Prism 8.0(GraphPad Software., San Diego, CA 92108, USA). P< 0.05 was considered as statistically significant.

### Ethics

Written informed consents were obtained from all patients; and, this study conformed to the principles of the Declaration of Helsinki were approved by the investigational review board of the Sichuan Provincial People's Hospital.

## References

[1] Dichgans M, Pulit SL, Rosand J. Stroke genetics: discovery, biology, and clinical applications. Lancet Neurol. 2019; 18:587–99. 10.1016/S1474-4422(19)30043-2 30975520

[2] Wu S, Wu B, Liu M, Chen Z, Wang W, Anderson CS, Sandercock P, Wang Y, Huang Y, Cui L, Pu C, Jia J, Zhang T, et al, and China Stroke Study Collaboration. Stroke in China: advances and challenges in epidemiology, prevention, and management. Lancet Neurol. 2019; 18:394–405. 10.1016/S1474-4422(18)30500-3 30878104

[3] Li Z, Jiang Y, Li H, Xian Y, Wang Y. China’s response to the rising stroke burden. BMJ. 2019; 364:l879. 10.1136/bmj.l879 30819725 PMC6394375

[4] Fournier LE, Zhang X, Bonojo E, Love M, Sanner J, Cooksey G, Hinojosa E, Okpala MN, Savitz SI, Sharrief AZ. Predictors of Post-Stroke Depression in Ischemic Stroke Patients using the Patient Health Questionnaire-9. Stroke. 2019; 50:A118–A118. 10.1161/str.50.suppl_1.118 PMC7243614 31941579

[5] Cai W, Mueller C, Li YJ, Shen WD, Stewart R. Post stroke depression and risk of stroke recurrence and mortality: A systematic review and meta-analysis. Ageing Res Rev. 2019; 50:102–09. 10.1016/j.arr.2019.01.013 30711712

[6] Villa RF, Ferrari F, Moretti A. Post-stroke depression: mechanisms and pharmacological treatment. Pharmacol Ther. 2018; 184:131–44. 10.1016/j.pharmthera.2017.11.005 29128343

[7] Ghyselinck NB, Duester G. Retinoic acid signaling pathways. Development. 2019; 146:dev167502. 10.1242/dev.167502 31273085 PMC6633611

[8] Oliveira LM, Teixeira FME, Sato MN. Impact of Retinoic Acid on Immune Cells and Inflammatory Diseases. Mediators Inflamm. 2018; 2018:3067126. 10.1155/2018/3067126 30158832 PMC6109577

[9] Lengerke C, Wingert R, Beeretz M, Grauer M, Schmidt AG, Konantz M, Daley GQ, Davidson AJ. Interactions between Cdx genes and retinoic acid modulate early cardiogenesis. Dev Biol. 2011; 354:134–42. 10.1016/j.ydbio.2011.03.027 21466798 PMC3502019

[10] Ratajska A, Złotorowicz R, Błazejczyk M, Wasiutyñski A. Coronary artery embryogenesis in cardiac defects induced by retinoic acid in mice. Birth Defects Res A Clin Mol Teratol. 2005; 73:966–79. 10.1002/bdra.20200 16323158

[11] Liu Y, Chen H, Mu D, Li D, Zhong Y, Jiang N, Zhang Y, Xia M. Association of serum retinoic acid with risk of mortality in patients with coronary artery disease. Circ Res. 2016; 119:557–63. 10.1161/CIRCRESAHA.116.308781 27323773

[12] Tu WJ, Qiu HC, Zhang Y, Cao JL, Wang H, Zhao JZ, Liu Q, Zeng X. Lower serum retinoic acid level for prediction of higher risk of mortality in ischemic stroke. Neurology. 2019; 92:e1678–87. 10.1212/WNL.0000000000007261 30850446

[13] Zhang Y, Kong F, Crofton EJ, Dragosljvich SN, Sinha M, Li D, Fan X, Koshy S, Hommel JD, Spratt HM, Luxon BA, Green TA. Transcriptomics of Environmental Enrichment Reveals a Role for Retinoic Acid Signaling in Addiction. Front Mol Neurosci. 2016; 9:119. 10.3389/fnmol.2016.00119 27899881 PMC5110542

[14] Zhang Y, Crofton EJ, Smith TE, Koshy S, Li D, Green TA. Manipulation of retinoic acid signaling in the nucleus accumbens shell alters rat emotional behavior. Behav Brain Res. 2019; 376:112177. 10.1016/j.bbr.2019.112177 31449909 PMC7359447

[15] Hu P, Wang Y, Liu J, Meng FT, Qi XR, Chen L, van Dam AM, Joëls M, Lucassen PJ, Zhou JN. Chronic retinoic acid treatment suppresses adult hippocampal neurogenesis, in close correlation with depressive-like behavior. Hippocampus. 2016; 26:911–23. 10.1002/hipo.22574 26860546

[16] Mawson AR, Xueyuan W. Breastfeeding, retinoids, and postpartum depression: a new theory. J Affect Disord. 2013; 150:1129–35. 10.1016/j.jad.2013.05.038 23816449

[17] Zeng Y, Li Y, Xia H, Wang S, Zhou J, Chen D. Retinoids, anxiety and peripartum depressive symptoms among Chinese women: a prospective cohort study. BMC Psychiatry. 2017; 17:278. 10.1186/s12888-017-1405-0 28764671 PMC5539642

[18] Whyte EM, Mulsant BH. Post stroke depression: epidemiology, pathophysiology, and biological treatment. Biol Psychiatry. 2002; 52:253–64. 10.1016/S0006-3223(02)01424-5 12182931

[19] Li L, Li Y, Ji X, Zhang B, Wei H, Luo Y. The effects of retinoic acid on the expression of neurogranin after experimental cerebral ischemia. Brain Res. 2008; 1226:234–40. J 10.1016/j.brainres.2008.06.037 18602376

[20] Duan Z, Shan W, Du H, Xu M, Feng J, Qiu C, Ling Y. Association between serum retinoic acid levels and risk of post-stroke depression in patients with ischemic stroke. Asian J Psychiatr. 2019; 46:87–91. 10.1016/j.ajp.2019.09.038 31639555

[21] Hou L, Ding C, Chen Z, Liu Y, Shi H, Zou C, Zhang H, Lu Z, Zheng D. Serum Retinoic Acid Level and The Risk of Poststroke Cognitive Impairment in Ischemic Stroke Patients. J Stroke Cerebrovasc Dis. 2019; 28:104352. 10.1016/j.jstrokecerebrovasdis.2019.104352 31501037

[22] Baccaro A, Wang YP, Candido M, Conforto AB, Brunoni AR, Leite CD, Busatto Filho G, Lotufo PA, Benseñor IM, Goulart AC. Post-stroke depression and cognitive impairment: study design and preliminary findings in a Brazilian prospective stroke cohort (EMMA study). J Affect Disord. 2019; 245:72–81. 10.1016/j.jad.2018.10.003 30368073

[23] Hackett ML, Pickles K. Part I: frequency of depression after stroke: an updated systematic review and meta-analysis of observational studies. Int J Stroke. 2014; 9:1017–25. 10.1111/ijs.12357 25117911

[24] Kapoor A, Lanctot KL, Bayley M, Herrmann N, Murray BJ, Swartz RH. Screening for post-stroke depression and cognitive impairment at baseline predicts long-term patient-centered outcomes after stroke. J Geriatr Psychiatry Neurol. 2019; 32:40–48. 10.1177/0891988718819859 30793663

[25] Paolucci S, Iosa M, Coiro P, Venturiero V, Savo A, De Angelis D, Morone G. Post-stroke Depression Increases Disability More Than 15% in Ischemic Stroke Survivors: A Case-Control Study. Front Neurol. 2019; 10:926. 10.3389/fneur.2019.00926 31507525 PMC6718567

[26] Gu Y, Zhu Z, Luan X, He J. Vitamin D status and its association with season, depression in stroke. Neurosci Lett. 2019; 690:99–105. 10.1016/j.neulet.2018.09.046 30261231

[27] Almeida OP, Marsh K, Alfonso H, Flicker L, Davis TM, Hankey GJ. B-vitamins reduce the long-term risk of depression after stroke: the VITATOPS-DEP trial. Ann Neurol. 2010; 68:503–10. 10.1002/ana.22189 20976769

[28] Zhang W, Wang W, Kuang L. The relation between insulin-like growth factor 1 levels and risk of depression in ischemic stroke. Int J Geriatr Psychiatry. 2018; 33:e228–33. 10.1002/gps.4774 28833493

[29] Kang HJ, Bae KY, Kim SW, Kim JT, Park MS, Cho KH, Kim JM. Effects of interleukin-6, interleukin-18, and statin use, evaluated at acute stroke, on post-stroke depression during 1-year follow-up. Psychoneuroendocrinology. 2016; 72:156–60. 10.1016/j.psyneuen.2016.07.001 27428088

[30] Yang RR, Lu BC, Li T, Du YF, Wang X, Jia YX. The relationship between high-sensitivity C-reactive protein at admission and post stroke depression: a 6-month follow-up study. Int J Geriatr Psychiatry. 2016; 31:231–39. 10.1002/gps.4315 26042821

[31] Tang CZ, Zhang YL, Wang WS, Li WG, Shi JP. Elevated serum levels of neopterin at admission predicts depression after acute ischemic stroke: a 6-month follow-up study. Mol Neurobiol. 2016; 53:3194–204. 10.1007/s12035-015-9220-4 26041659

[32] Kim JM, Kang HJ, Kim JW, Bae KY, Kim SW, Kim JT, Park MS, Cho KH. Associations of Tumor Necrosis Factor-α and Interleukin-1β Levels and Polymorphisms with Post-Stroke Depression. Am J Geriatr Psychiatry. 2017; 25:1300–08. 10.1016/j.jagp.2017.07.012 28844626

[33] Tu WJ, Qiu HC, Liu Q, Li X, Zhao JZ, Zeng X. Decreased level of irisin, a skeletal muscle cell-derived myokine, is associated with post-stroke depression in the ischemic stroke population. J Neuroinflammation. 2018; 15:133. 10.1186/s12974-018-1177-6 29720216 PMC5932807

[34] Tu WJ, Dong X, Zhao SJ, Yang DG, Chen H. Prognostic value of plasma neuroendocrine biomarkers in patients with acute ischaemic stroke. J Neuroendocrinol. 2013; 25:771–78. 10.1111/jne.12052 23701638

[35] Jones KA, Thomsen C. The role of the innate immune system in psychiatric disorders. Mol Cell Neurosci. 2013; 53:52–62. 10.1016/j.mcn.2012.10.002 23064447

[36] Spalletta G, Bossù P, Ciaramella A, Bria P, Caltagirone C, Robinson RG. The etiology of poststroke depression: a review of the literature and a new hypothesis involving inflammatory cytokines. Mol Psychiatry. 2006; 11:984–91. 10.1038/sj.mp.4001879 16894392

[37] Bakdash G, Vogelpoel LT, van Capel TM, Kapsenberg ML, de Jong EC. Retinoic acid primes human dendritic cells to induce gut-homing, IL-10-producing regulatory T cells. Mucosal Immunol. 2015; 8:265–78. 10.1038/mi.2014.64 25027601

[38] Collins CB, Aherne CM, Kominsky D, McNamee EN, Lebsack MD, Eltzschig H, Jedlicka P, Rivera-Nieves J. Retinoic acid attenuates ileitis by restoring the balance between T-helper 17 and T regulatory cells. Gastroenterology. 2011; 141:1821–31. 10.1053/j.gastro.2011.05.049 22027263 PMC3684415

[39] Wang C, Kang SG, HogenEsch H, Love PE, Kim CH. Retinoic acid determines the precise tissue tropism of inflammatory Th17 cells in the intestine. J Immunol. 2010; 184:5519–26. 10.4049/jimmunol.0903942 20400707 PMC3009589

[40] Najjar S, Pearlman DM, Devinsky O, Najjar A, Zagzag D. Neurovascular unit dysfunction with blood-brain barrier hyperpermeability contributes to major depressive disorder: a review of clinical and experimental evidence. J Neuroinflammation. 2013; 10:142. 10.1186/1742-2094-10-142 24289502 PMC4220803

[41] Kong L, Wang Y, Wang XJ, Wang XT, Zhao Y, Wang LM, Chen ZY. Retinoic acid ameliorates blood-brain barrier disruption following ischemic stroke in rats. Pharmacol Res. 2015; 99:125–36. 10.1016/j.phrs.2015.05.014 26066585

[42] Nabavi SF, Dean OM, Turner A, Sureda A, Daglia M, Nabavi SM. Oxidative stress and post-stroke depression: possible therapeutic role of polyphenols? Curr Med Chem. 2015; 22:343–51. 10.2174/0929867321666141106122319 25386821

[43] Ahlemeyer B, Bauerbach E, Plath M, Steuber M, Heers C, Tegtmeier F, Krieglstein J. Retinoic acid reduces apoptosis and oxidative stress by preservation of SOD protein level. Free Radic Biol Med. 2001; 30:1067–77. 10.1016/S0891-5849(01)00495-6 11369496

[44] Achan V, Tran CT, Arrigoni F, Whitley GS, Leiper JM, Vallance P. all-trans-Retinoic acid increases nitric oxide synthesis by endothelial cells: a role for the induction of dimethylarginine dimethylaminohydrolase. Circ Res. 2002; 90:764–69. 10.1161/01.RES.0000014450.40853.2B 11964368

[45] Aggarwal A, Gaur V, Kumar A. Nitric oxide mechanism in the protective effect of naringin against post-stroke depression (PSD) in mice. Life Sci. 2010; 86:928–35. 10.1016/j.lfs.2010.04.011 20433854

[46] Brooker SM, Gobeske KT, Chen J, Peng CY, Kessler JA. Hippocampal bone morphogenetic protein signaling mediates behavioral effects of antidepressant treatment. Mol Psychiatry. 2017; 22:910–19. 10.1038/mp.2016.160 27698430 PMC5378681

[47] Shen H, Luo Y, Kuo CC, Deng X, Chang CF, Harvey BK, Hoffer BJ, Wang Y. 9-Cis-retinoic acid reduces ischemic brain injury in rodents via bone morphogenetic protein. J Neurosci Res. 2009; 87:545–55. 10.1002/jnr.21865 18803283 PMC2628966

[48] Larange A, Cheroutre H. Retinoic acid and retinoic acid receptors as pleiotropic modulators of the immune system. Annu Rev Immunol. 2016; 34:369–94. 10.1146/annurev-immunol-041015-055427 27168242

[49] Stebbins MJ, Lippmann ES, Faubion MG, Daneman R, Palecek SP, Shusta EV. Activation of RARα, RARγ, or RXRα Increases Barrier Tightness in Human Induced Pluripotent Stem Cell-Derived Brain Endothelial Cells. Biotechnol J. 2018; 13:1700093. 10.1002/biot.201700093 28960887 PMC5796863

[50] Walters BJ, Josselyn SA. Retinoic acid receptor plays both sides of homeostatic plasticity. Proc Natl Acad Sci USA. 2019; 116:6528–30. 10.1073/pnas.1902400116 30872478 PMC6452678

[51] Chiang MY, Misner D, Kempermann G, Schikorski T, Giguère V, Sucov HM, Gage FH, Stevens CF, Evans RM. An essential role for retinoid receptors RARbeta and RXRgamma in long-term potentiation and depression. Neuron. 1998; 21:1353–61. 10.1016/S0896-6273(00)80654-6 9883728

[52] Ke Q, Li R, Cai L, Wu SD, Li CM. Ro41-5253, a selective antagonist of retinoic acid receptor α, ameliorates chronic unpredictable mild stress-induced depressive-like behaviors in rats: involvement of regulating HPA axis and improving hippocampal neuronal deficits. Brain Res Bull. 2019; 146:302–09. 10.1016/j.brainresbull.2019.01.022 30711623

[53] Machado-Pereira M, Santos T, Ferreira L, Bernardino L, Ferreira R. Intravenous administration of retinoic acid-loaded polymeric nanoparticles prevents ischemic injury in the immature brain. Neurosci Lett. 2018; 673:116–21. 10.1016/j.neulet.2018.02.066 29518539

[54] Plane JM, Whitney JT, Schallert T, Parent JM. Retinoic acid and environmental enrichment alter subventricular zone and striatal neurogenesis after stroke. Exp Neurol. 2008; 214:125–34. 10.1016/j.expneurol.2008.08.006 18778705 PMC2896452

[55] Harvey BK, Shen H, Chen GJ, Yoshida Y, Wang Y. Midkine and retinoic acid reduce cerebral infarction induced by middle cerebral artery ligation in rats. Neurosci Lett. 2004; 369:138–41. 10.1016/j.neulet.2004.07.086 15450683

[56] Cai W, Wang J, Hu M, Chen X, Lu Z, Bellanti JA, Zheng SG. All trans-retinoic acid protects against acute ischemic stroke by modulating neutrophil functions through STAT1 signaling. J Neuroinflammation. 2019; 16:175. 10.1186/s12974-019-1557-6 31472680 PMC6717357

[57] WHO MONICA Project Principal Investigators. The World Health Organization MONICA Project (monitoring trends and determinants in cardiovascular disease): a major international collaboration. J Clin Epidemiol. 1988; 41:105–14. 10.1016/0895-4356(88)90084-4 3335877

[58] Brott T, Marler JR, Olinger CP, Adams HP Jr, Tomsick T, Barsan WG, Biller J, Eberle R, Hertzberg V, Walker M. Measurements of acute cerebral infarction: lesion size by computed tomography. Stroke. 1989; 20:871–75. 10.1161/01.STR.20.7.871 2749847

[59] Adams HP Jr, Bendixen BH, Kappelle LJ, Biller J, Love BB, Gordon DL, Marsh EE 3rd. Classification of subtype of acute ischemic stroke. Definitions for use in a multicenter clinical trial. TOAST. Trial of Org 10172 in Acute Stroke Treatment. Stroke. 1993; 24:35–41. 10.1161/01.STR.24.1.35 7678184

[60] Bonita R, Beaglehole R. Recovery of motor function after stroke. Stroke. 1988; 19:1497–500. 10.1161/01.STR.19.12.1497 3201508

[61] Robinson RG, Jorge RE. Post-stroke depression: a review. Am J Psychiatry. 2016; 173:221–31. 10.1176/appi.ajp.2015.15030363 26684921

[62] Zheng YP, Zhao JP, Phillips M, Liu JB, Cai MF, Sun SQ, Huang MF. Validity and reliability of the Chinese Hamilton depression rating scale. Br J Psychiatry. 1988; 152:660–64. 10.1192/bjp.152.5.660 3167442

[63] Bjelland I, Dahl AA, Haug TT, Neckelmann D. The validity of the Hospital Anxiety and Depression Scale. An updated literature review. J Psychosom Res. 2002; 52:69–77. 10.1016/S0022-3999(01)00296-3 11832252

[64] Liu Y, Chen H, Wang J, Zhou W, Sun R, Xia M. Association of serum retinoic acid with hepatic steatosis and liver injury in nonalcoholic fatty liver disease. Am J Clin Nutr. 2015; 102:130–37. 10.3945/ajcn.114.105155 25948673

